# Experiences of working as a clinical nurse while pregnant during the coronavirus disease-2019 pandemic: a qualitative study

**DOI:** 10.1186/s12912-025-02764-z

**Published:** 2025-02-03

**Authors:** Lin Ye Wu, Wing Fai Yeung, Yao Lin Pei, Ling Xi Chen, Meng Qi Li, Jia Yin Ruan

**Affiliations:** 1 Medical Care Department, Zhejiang Hospital, Hangzhou, Zhejiang China; 2 School of Nursing, The Hong Kong Polytechnic University, Hung Hom, Kowloon, Hong Kong China; 3 School of Nursing, The University of Texas at Austin, Austin, TX USA; 4 School of Nursing, Zhejiang Chinese Medical University, Hangzhou, Zhejiang China; 5 Kiang Wu Nursing College of Macau, Est. Repouso No.35 Macau, Macao, China; 6 New York University Rory Meyers College of Nursing, New York City, NY USA

**Keywords:** Experiences, Pregnant, Nurse, COVID-19 pandemic, Qualitative research

## Abstract

**Background:**

Working as a pregnant clinical nurse might experience a range of challenges, such as significant anatomical and physiological changes as well as emotional and cognitive changes. That might be particularly obvious under the historical background of coronavirus disease-2019 (COVID-19) pandemic. However, a dearth of studies has explored the experiences of working as a pregnant nurse during this special period. This study aimed to explore the experiences of working as a clinical nurse while pregnant during the COVID-19 pandemic.

**Methods:**

A descriptive qualitative design was selected. Purposive sampling, combined with maximum variation strategy and snowball sampling, were utilized to identify and select participants from tertiary-teaching hospitals, specialized hospitals, and community hospitals in Zhejiang Province, southeastern China. Online semi-structured individual interviews were used to collect data, and conventional content analysis was used to analyze the data.

**Results:**

Eleven Chinese nurses with a mean age of 31.8 years, ranging from 26 to 40 years, participated in this study. Four themes and twelve subthemes emerged: (1) still adhering to work as a clinical nurse despite being pregnant during the pandemic; (2) working during pregnancy under pandemic is still an ordinary nurse; (3) still staying in the special life phase as a pregnant mother; and (4) growth and gains as pregnant mother.

**Conclusion:**

The pregnant clinical nurses suffered from various changes and difficulties during the pandemic. Managers, occupational health and other health system leaders, and policymakers should be aware of the importance of establishing a work environment that guarantees safe continued pregnancy. Future studies should focus on the establishment of specific guidelines and manuals regarding how pregnant nurses worked, as well as the development of self-protection interventions during pregnancy. Moreover, research on moral stigma and bullying in nursing during pregnancy deserves further exploration.

**Clinical trial number:**

Not applicable.

## Background

Nursing, both an art and science, is generally considered a female-dominated profession. This situation is particularly obvious in East Asian countries compared with Western countries (e.g., 96% female nurses in Korea and China versus 90.4% in the United States of America) [1–3]. According to evidence, 49.2% of nurses were in their childbearing age (18–49 years) [3], and this percentage was markedly higher in East Asian countries (e.g., 85.5% in China) [1]. This indicates the high likelihood that nurses may intend to become pregnant.

Accumulating evidence has confirmed that clinical registered nurses face several difficulties. Nurses may bear excessive workload, work overtime [4], have irregular night shifts [5], experience frequent occupational exposure to chemical, physical, and biological hazards (e.g., 52.1% being exposed to blood or body fluids) [6], and suffer from work violence and complex interpersonal relationships. These factors result in physical health and mental health impairment to varying degrees. An estimated 77.2% of nurses had work-related musculoskeletal diseases [7], and 73.8% of those experienced general chronic pain [8]. Approximately 30% of nurses had depression, while 41.2% reported anxiety [9]. During pregnancy nurses might experience a range of symptoms (e.g., significant anatomical and physiological changes [10], physical changes [11], fatigue, insomnia [12], emotional and cognitive changes [12, 13]). Therefore, working as a pregnant clinical nurse may be associated with additional challenges (e.g., three-fold increased risk of miscarriage compared with females working in other industries) [14]. Notably, these negative consequences towards nurses were more severe during the coronavirus disease 2019 (COVID-19) pandemic [15, 16].

COVID-19 is a highly transmissible disease presenting major challenges to the global healthcare system [17]. From its occurrence in December 2019, until December 2024, a total of more than 777 million cases of COVID-19 were reported, including 99.4 million cases in China [18]. Nurses played a significant role in maintaining the healthcare systems active during the pandemic [19] by caring for patients with COVID-19 [20] and participating in the general public healthcare. Based on the dynamic zero-COVID-19 policy (from April 2020 to December 2022) [21, 22], Chinese nurses were also trained and prepared to conduct nucleic acid detection at their workplace or transferred to areas affected by severe epidemics. Furthermore, since the termination of the dynamic zero-COVID-19 policy (December 2022), clinical nurses (including pregnant nurses) in mainland China suffered from a huge care burden towards a rapidly surging number of infected individuals within a very short time frame [23].

Interestingly, during the implementation of the dynamic zero-COVID-19 policy and after its termination, a number of pregnant clinical nurses continued to work in the frontline. This fact raises the following questions. Which factors facilitated the continuation of work in clinical practice for those pregnant nurses? What was the experience of working as a pregnant clinical nurse? What are the needs of pregnant clinical nurses? What challenges did they encounter during this particular period? How did pregnant clinical nurses handle these difficulties? These questions could be answered by revealing the experiences of those pregnant clinical nurses who continued to work during the COVID-19 pandemic. The findings may promote the development of need-based interventions for pregnant nurses, provide targeted support, and create a safe environment for pregnant nurses and their fetuses. These measures may lead to improvement in physical and mental health status and turnover rate reduction.

In recent years, the experiences of working as pregnant nurses have received considerable research attention. A grounded theory study from the United States explored how nurses incorporated pregnancy with professional nursing employment in early and late stages [24]. A descriptive qualitative study from the same country described the occupational hazards and risks during pregnancy [2]. Similarly, research from Korea found that pregnant nurses were exposed to risky work environments [25]. However, none of the three studies depicted continued shift work during pregnancy [26], nor did they explore related experiences during the unique circumstances of the COVID-19 pandemic. Meanwhile, the methodological quality of these studies was not high, with only 1 study receiving full marks [25, 26]. Although a recent qualitative study from India explored the challenges and experiences of pregnant health care professionals during the COVID-19 pandemic, most participants were doctors and only 5 were staff nurses [27]. More importantly, the findings from the aforementioned four studies, conducted in the U.S., Korea, and India, may not be able to provide a direct and comprehensive understanding of the experiences of working as a pregnant clinical nurse in China. This might be due to differences in culture, healthcare system, and socioeconomic status. Therefore, the aim of this study was to explore the experiences of working as pregnant clinical nurses during the COVID-19 pandemic period in China.

## Methods

A descriptive qualitative design was adopted for this study [28]. Descriptive qualitative research, which is underpinned by general tenets from naturalistic inquiry (e.g., no manipulation of variables) [29], focuses on what, who, and where of an event or experience and aims to offer a direct description of the event or the experience in daily life [28]. Due to the nature of qualitative study, the clinical trial number is not applicable.

### Sampling and recruitment

Purposive sampling, combined with snowball sampling and maximum variation strategy, were utilized to identify and select participants [30, 31]. The maximum variation was pursued in terms of age, working department, years of experience as a registered nurse, the number of pregnancies, and parity. Participants were recruited from seven tertiary-teaching hospitals, specialized hospitals, and community hospitals in Zhejiang Province, southeastern China. Inclusion criteria were: (1) registered nurses who provided direct patient care; (2) clinical nurses with experiences of working while pregnant during the COVID-19 pandemic [32]; (3) nurses who were able to speak and write Chinese; and (4) nurses who were willing to participate in the study and signed the consent form. Of note, nurses who had a psychiatric disease were excluded.

Potential eligible participants were approached based on the personal network of the researchers. Following an agreement to participate, an interview was scheduled. Twelve nurses approached, including nine who were familiar with the researchers before the interview, three were recommended by two participants after their own interviews, and one refused due to time conflict.

### Data collection

Since April 2023 to December 2023, semi-structured individual interviews though WeChat (a Chinese mobile messaging app and social media platform) were conducted by LW and JR, both have rich experience in qualitative interviews [33–35]. An interview guide with open-ended questions was created based on literature review [2, 25], and personal experience (Table 1). As the research proceeded, the interview questions became more structured.

**Table 1:** Interview guide

1.Please share with me your positive experiences of working as a nurse while pregnant during the COVID-19 pandemic.
2.Please share with me your negative experiences of working as a nurse while pregnant during the COVID-19 pandemic.
3.Please share with me any impressive events that occurred during the COVID-19 pandemic while working as a clinical pregnant nurse.
4.Please share with me any challenges or difficulties that you faced as a pregnant clinical nurse during the COVID-19 pandemic.
5.Please share with me any needs while being pregnant clinical nurse during the COVID-19 pandemic.

During the interview, participants were encouraged to express themselves openly. All interviews were audio-recorded, transcribed by LW and checked word for word by JR. The mean time of interviews was 82 min (range: 44–193 min). The details of the interview interactions, environment, and non-verbal signs were recorded immediately after each interview.

### Data analysis

Data analysis was conducted manually and carried out concurrently with data collection, using conventional content analysis methods by LW [36]. The language used for data analysis was Chinese, and English was used for reporting quotes, subthemes, and themes. First, LW read and reread the transcription of each participant several times to obtain an overall understanding of the data. Subsequently, the coder independently coded the transcripts line-by-line to identify any narrative data that are related to the experiences of working as a pregnant clinical nurse during the COVID-19 pandemic. For example, for one sentence from the transcript one: “At that time, I was not allowed to take leave because our department was applying for the elderly specialist nurse base at that period.” LW coded this as: “Not allowed to take leave due to department needs.” Next, the coder grouped the coding units based on shared characteristics and presented the working experiences as a pregnant clinical nurse in a more abstract manner. An example illustrates this: the coding units “not allowed to take leave while pregnant during COVID-19 pandemic time due to lacking nurses” and “applying for leave being troublesome” were grouped together under the theme “being particularly difficult to ask for leave during special period.” Thereafter, similar coding units with a higher abstract level were extracted and clustered into subthemes and themes through an interactive and inductive process. During the analysis process, LW discussed with a senior qualitative researcher (JR) regarding initial analysis results, subthemes and themes that were established during further discussion within the research team. Modifying the subtheme 4.1 from “being a better person” to “being a mature person” took as a case. Data saturation was obtained after the 8th interview when similar responses started to be heard repeatedly, and no emergence of new subthemes or themes associated with experiences of working as a clinical nurse while pregnant during the COVID-19 pandemic were noted [37].

### Ethical considerations

This study was approved by the Research Ethics Committee of Zhejiang Hospital (approval number:2023(03k)). Participants were informed of the purpose of the study. They understood that their participation was voluntary, and they could withdraw from the interview at any time without penalty. Confidentiality and anonymity were guaranteed by the researchers.

### Rigor

Several strategies were used to enhance the reliability of the qualitative findings [38–40]. Firstly, the researchers encouraged participants to speak freely to gain authenticity. Secondly, credibility was established through peer debriefing, in which the researchers addressed any disagreement or ambiguities on methodological approaches or data analysis [38, 40]. Thirdly, the researchers maintained reflective journals continuously throughout the study period. For instance, LW recorded her personal experiences of working as a pregnant clinical nurse during the COVID-19 before data collection (e.g., I was unwilling to continue work as a pregnant clinical nurse after the termination of the dynamic zero-COVID-19 policy, but did not have a choice due to shortage of nurses during that time) and attempted to see the studied phenomenon with fresh eyes and understand the experience wholly.

## Results

A total of 11 clinical nurses with a mean age of 31.8 years which ranges from 26 to 40 years participated in the qualitative interviews. Table 2 presents the sociodemographic and characteristics of the participants.

**Table 2:** Sociodemographic and characteristics of the participants

No.	Age	Years of experience as a nurse (weeks)	Education level	Work setting	Work setting during the pregnancy	Shift	Night shift length (month)	Maternity leave length^*^ (days)	Leave length during the pregnancy (weeks)	Number of pregnancies/parities	Time of childbirth during the COVID
P1	31	55	master	geriatric	geriatric	nightshift and day shift	7	128	4	1/1	2020.07
P2	33	56	master	nephrology	nephrology	day shift		188	12	2/2	2022.06
P3	31	56	master	ICU^*^/palliative care	ICU^*^/palliative care	nightshift and day shift	7	188	1	2/2	2021.01 2022.12
P4	28	128	bachelor	palliative care	community outpatient	nightshift and day shift	7	128	1	2/2	2022.12
P5	26	46	bachelor	urinary surgery	fever clinic	day shift	NA	158	12	1/1	2022.11
P6	33	118	bachelor	operation	trauma unit	night shift and day shift	5	128	0	2/2	2020.07
P7	32	118	bachelor	ICU^*^	ICU^*^	day shift	NA	218	24	4/2	2020.10 2022.11
P8	40	275	bachelor	orthopaedic department	traditional Chinese medicine outpatient	day shift	NA	173	16	3/1	2021.11
P9	29	51	master	cardiology	cardiology	day shift	NA	158	8	1/1	2022.11
P10	33	112	bachelor	hematology	hematology	day shift	NA	173	20	1/1	2022.11
P11	28	76	bachelor	department of neurosurgery/palliative care	outpatient palliative care	night shift and day shift	7	188	0	2/2	2021.02 2023.03

Note: Maternity leave length: According to the Population and Family Planning Regulations of Zhejiang Province, the maternity leave length was 128 days, for difficult childbirth to increase by 15 days, for every additional baby born, an additional 15 days of prenatal checkups are required. And starting from December 25, 2021, the maternity leave for the first child will be changed from 128 days to 158 days, for the second and third children to 188 days; NA = Not Applicable; ICU = Intensive Care Unit

Four themes and twelve subthemes related to the pregnant clinical nurses’ experiences during the COVID-19 pandemic emerged (Table 3). Theme 1: still adhering to work as a clinical nurse despite being pregnant during the pandemic; Theme 2: working during pregnancy under pandemic is still an ordinary nurse; Theme 3: still staying in the special life phase as a pregnant mother; and Theme 4: growth and gains as pregnant mother.

**Table 3:** Themes and subthemes

Theme	Subtheme
Still adhering to work as a clinical nurse despite being pregnant during the pandemic	Being particularly difficult to ask for leave during special period
	Not dare to take time off as society person
	Not willing to leave by themselves
Working during pregnancy under pandemic is still an ordinary nurse	Still doing the same work content as other nonpregnant nurses
	Still arranging the same job requirements as other nonpregnant nurses
	Still being assigned COVID-19 related care as other nonpregnant nurses
Still staying in the special life phase as a pregnant mother	Being disturbed by symptoms induced by pregnancy
	Being very cautious to protect themselves and their upcoming babies
	Becoming sensitive, vulnerable and nervous
	Being confused about their pregnancy
Growth and gains as pregnant mother	Being a mature person
	Obtaining support from others

### Still adhering to work as a clinical nurse despite being pregnant during the pandemic

Nurses working during the pandemic faced medical supply shortages and were more likely to experience physical and mental effects. Nevertheless, some pregnant nurses continued to work for several reasons.

#### Being particularly difficult to ask for leave during special period

This theme reflects the difficulty of clinical nurses asking for leave during the special period, which was mainly related to two causes.

One cause is about nursing shortage phenomenon in China, which was particularly severe during the COVID-19 pandemic period when the workload became specifically heavy. The need to provide care and manage patients with COVID-19 in other cities in the same province or in different provinces in China further aggravated this shortage. Under these circumstances, it became especially difficult to ask for sick leave or maternity leave earlier. Therefore, those nurses had to continue working despite their pregnancy.

*“You could not ask for leave at that time (COVID-19 pandemic time)*,* the hospital would not approve of your leave.” (P11)*.

Sometimes only a few nurses with certain traits and abilities were available in corresponding departments, those with a master’s degree in clinical practice. The requirement to complete certain work, such as nursing research tasks, made it difficult for those pregnant nurses to ask for leave earlier.

*“The time to submit the research project was relatively tight. Despite being pregnant*,* I was not allowed to leave earlier.” (P2)*.

Another reason was related to the complicated, inflexible, and cumbersome application procedure for leave. This made some participants choose to continue working rather than ask for leave. As one participant stated:

*“The process for pregnant women to request leave is very complicated. If you need to*,* you should get a diagnosis from a physician first*,* get stamped by a required department and lastly be approved by the nursing department. However*,* you know*,* the validity of the leave proof only lasts for 1 week!” (P3)*.

#### Not dare to take time off as a society person

As a society person, apart from the role of being a nurse, pregnant nurses also held other identities and need to shoulder family responsibilities, such as spending more time accompanying upcoming babies as much as possible. To request for leave during pregnancy could potentially reduce the duration of the maternity leave. Hence, some participants selected to continue working for as long as possible.

*“If you ask for leave*,* then you have to deduct your maternity leave time.” (P3)*.

Maintaining a certain income to support their nuclear family and/or their natal family was another responsibility of clinical nurses. To achieve this, some participants continued working during their pregnancy.

*“The economic pressure was heavy*,* and the mortgages needed to be paid. I did not dare to take leave. I need some economic support.” (P2)*.

Always afraid of being labelled as someone not dedicated to the nursing profession but always thinking about her own needs first, some participants continued to work even though sacrificed their inner demands. That might highly occur within the workplace which was influenced by the culture taking pregnant nurses who still work for granted.

*“No one takes a rest during pregnancy. It’s like a tradition*,* everyone was at work with a big belly*,* and nobody took a rest*,* unless you were preventing miscarriages.” (P6)*.

#### Not willing to leave by themselves

This subtheme revealed three reasons that facilitated participants actively stay in their work. “Still competent to nursing work” is one reason. This might be associated with participants’ capable physical and mental status, and reduced workload when being looked after.

*“My health status was fine. My pregnancy reaction was minor. Thus*,* I did not consider taking a rest.” (P2)*.

Another participant who was looked after by her nurse manager and transferred to the outpatient department with a relatively light workload said,

*“The work was light and easy*,* so I did not choose to rest. If I were still in the ward (urinary surgery)*,* I would definitely ask for leave and rest earlier.” (P5)*.

“Treating work as a way to connect with society” is another cause. Some participants expressed that they would feel lonely and uncomfortable if they started to stay at home from an earlier stage of pregnancy. This was often reported by nurses who only lived with their husband, or together with a relative of their husband. When the husband was absent, being alone at home would lead to the emergence of negative feelings.

*“If not at work*,* I would definitely feel uncomfortable and lonely*,* always being with my unfamiliar mother-in-law. I may not gain warm feelings from the insufficient family support system.” (P1)*.

### Working during pregnancy under pandemic is still an ordinary nurse

Nurses who continued to work during pregnancy were regarded as manpower resource similar to other ordinary nurses. The pregnant nurse may play various roles assigned by her head nurse based on the needs of the department and take responsibility for tasks in the same manner as other non-pregnant nurses, no matter in job content, or in work requirements.

#### Still doing the same work content as other nonpregnant nurses

Despite being pregnant, nurses were still required to handle the same workload as other non-pregnant clinical nurses. As one participant said,

*“At that time*,* I was close to giving birth and had a very large belly. I still suctioned phlegm for patients while wearing an N95 mask and a face shield. It was really exhausting!” (P3)*.

If the patient being cared for was in a critical state, the pregnant charge nurses were expected to handle emergencies as other non-pregnant nurses. One participant recalled her rescue experience during pregnancy:

“*Although we were pregnant*,* it did not mean that we refrained from participating in rescues. Instead*,* we still actively participated as other nurses did.*” *(P8)*.

Similarly, pregnant nurses were anticipated to take care of patients with infectious diseases.

“*One patient has acquired immunodeficiency syndrome*,* and his indwelling needle was bad. I wore a glove and injected it by myself. Although pregnant*,* I felt embarrassed to ask others to help me care for these patients.*” *(P6)*.

#### Still arranging the same job requirements as other nonpregnant nurses

This subtheme indicated that working hours, the necessity of working on the night shift, and the requirement for high-quality work were the same among pregnant and non-pregnant nurses. Regarding working hours, occasionally, pregnant nurses even worked overtime due to the excessive workload.

“*Since it’s a newly opened department*,* everyone is extremely busy. Even though I’m pregnant*,* I still work overtime until 6 o’clock*,* while the regular off-duty time is 5 o’clock. ” (P10)*.

The labor policy states that nurses should work on the night shift until 7 months of pregnancy. Nonetheless, according to a few participants, this continues to bring huge work burden to pregnant nurses.

“*I worked day shift and night shift for first 7 pregnant months; I was very tired with a big belly. Our night shift was not easy*,* you had many things to handle*,* not the one you just sit there and let time go by.*” *(P6)*.

Last but not least, there were some quality requirements, such as the medicine should be completely dissolved.

*“My head nurse would go to the trash can to check whether the antibiotics were completely dissolved. One day*,* because I hadn’t completely dissolved them*,* the head nurse rushed to the rest room and scolded me while I was still resting.” (P3)*.

#### Still being assigned COVID-19 related care as other nonpregnant nurses

There is an excessive COVID-19 related workload required to be handled. Those pregnant nurses were distributed the same type and volume as those with nonpregnant nurses. The pregnant nurses who worked in large general hospitals needed to do quarantine work. For example, one participant who dealt with the medical orders stated:

*“At that time*,* patients needed to collect nucleic acid once a week and pay their own expenses. We needed to handle the order and communicate with the doctor frequently about this matter.” (P3)*.

Others needed to manage the access control and register the turn-around time of patients, as well as check the nucleic acid reports of the patients and their families.

“*Our department arranged a door guard nurse*,* this nurse should check the results of the nucleic acid reports of the families and register the visitor.” (P7)*.

As the community nurses were at the grassroots level, they needed to undertake substantial work toward epidemic prevention, such as vaccination and COVID-19 tracing.

*“The vaccination nurse was shortage*,* so the pregnant nurse also needs to take vaccination to others after the daily work*,* even off the night shift.” (P4)*.

### Still staying in the special life phase as a pregnant mother

A pregnant nurse may experience uncomfortable symptoms or negative emotions during the pregnancy or the childbirth time.

#### Being disturbed by symptoms induced by pregnancy

Pregnancy may lead to various uncomfortable symptoms (e.g., heavy body and edema). In particular, for pregnant nurses in the third trimester, sitting or standing for a long time made them feel very uncomfortable.

*“In my third trimester of pregnancy*,* I was affected by many symptoms that made it difficult for me to get through a full day of work. Specifically*,* my body felt heavy*,* my heart was under significant strain*,* my legs were very swollen*,* and my feet became very heavy and painful about 1 or 2 hours before the end of my shift.” (P9)*.

Others mentioned that wearing protective masks during the pandemic caused them to feel anoxic and required oxygen uptake in their daily work.

*“During the epidemic period*,* I wear the mask more standard in daily work*,* and sometimes I felt hypoxia and need to take some oxygen.” (P11)*.

Moreover, impaired memory was another aspect. Pregnant nurses realized that, occasionally, they could not recall prior knowledge.

*“I really could not answer the question while being examined by nursing manager regarding blood transfusions.” (P10)*.

#### Being very cautious to protect themselves and their upcoming babies

Nurses who were working during pregnancy paid particular attention to risk exposure, such as physical injury, infections, chemical hazards, and radiation exposure. Almost all participants expressed their fear of being infected due to needle prick injuries and were very cautious while administering an injection to patients.

Some participants mentioned that they adopted measures to prevent infectious diseases, such as those caused by multiple drug-resistant bacteria or viruses. This phenomenon was particularly observed in the intensive care unit (ICU), emergency department, and trauma surgery unit. One participant from the ICU stated:

“*During pregnancy*,* I became particularly careful to take care of patients with scabies. I wore gloves and performed all the actions very carefully to avoid touching any of their belongings. More importantly*,* I washed my hands several times until my skin peeled.” (P3)*.

During the COVID-19 pandemic, some participants mentioned they were particularly cautious in their work.

“*Prior to the pandemic*,* sometimes*,* I didn’t wear a mask standardly due to discomfort. However*,* during the pandemic*,* I wore a mask not only standardly but also strictly (I didn’t dare to take off the mask during worktime)*,* especially during the period of pregnancy.” (P11)*.

Moreover, preventing exposure to radiation was another aspect that participants particularly focused on. Notably, those nurses remained confused regarding the usefulness of those protective measures.

“*I wear radiation resistant clothing*,* although I know it may not be useful*,* it feels better when worn.*” *(P1)*.

#### Becoming sensitive, vulnerable and nervous

During pregnancy, nurses often experienced emotional changes, consequently becoming vulnerable, sensitive, and nervous.

Considering the shortage of nurses, the absence of pregnant nurses would further aggravate the shortage of manpower in the department. A pregnancy for relatively younger nurses who entered the workforce (i.e., 1 month to 2 years of experience) could cause dissatisfaction to the head nurse or senior colleagues in the department. As a result, those young pregnant nurses became particularly sensitive to verbal and non-verbal feedback.

“*The head nurse asked the nurse with only 1 year of working experience: ‘do you really want this baby*,* there are other senior nurses who never got pregnant’. When I heard this*,* I just thought that the head nurse must dislike me*,* the one who was the novice nurse with only 2 years of working experiences but pregnant.*” *(P3)*.

In addition, in the healthcare system, the hierarchy of nurses was relatively low. Nurses were commonly disrespected by doctors and even other colleagues, such as caregivers. During pregnancy, nurses may become more sensitive and vulnerable to these disrespectful behaviors and may even cry.

*“I felt very sad*,* that she (the doctor) disliked and avoided me because of my slow walking motions during resuscitation and required me to hurry up*,* never took my actual pregnant fact with big belly into consideration.” (P1)*.

Besides, some participants mentioned they feared making mistakes as clinical nurses during pregnancy, leading them to being nervous.

*“I would check twice and be very careful at work*,* but I still made mistakes and was scolded by the head nurse. Consequently*,* I was very scared and was afraid of making mistakes later on.” (P3)*.

In addition, as the clinical work requires cooperation, the pregnant nurse may occasionally bring trouble to others. This may cause someone to feel guilt due to feelings of becoming a burden to others.

*“If you do not do your job well*,* it might bring trouble to other colleagues. I didn’t want to trouble others.” (P1)*.

Moreover, due to the pandemic, family members of some participants did not encourage them to continue working in hospitals as nurses because of the potential risk of infecting the entire family. That may increase the participants’ nervousness.

*“My husband thought that I might pose an infection risk of COVID-19 to the whole family due to being a pregnant nurse still working in the frontline. Thus*,* he asked me to ask for a leave.” (P3)*.

#### Being confused about their pregnancy

The nurses reported that they felt confused about their pregnancy during the pandemic, mainly in two aspects. The first aspect was related to the lack of knowledge and skills related to pregnancy and the upcoming baby care.

“*The test result of my folic acid index was abnormal. I wondered whether I should continue to take folic acid supplement even after taking it for 3 months. I inquired the physician and also checked information by myself.” (P8)*.

The second aspect was associated with confusion brought by COVID-19 and related factors, such as vaccinations. Due to the requirement of COVID-19 vaccinations by policy, some nurses were aware of the pregnancy at the time of the vaccination, while others found that they were pregnant after receiving the vaccinations. Most of them were confused regarding the potential influences of the vaccinations on their fetuses.

*“I was vaccinated at that time with the COVID-19 vaccination. Because I got pregnant after the vaccine*,* I was afraid it would affect my baby.” (P2)*.

In addition, many participants mentioned that they were confused about the impact of COVID-19 on pregnant women, fetuses, and newborns, as well as the knowledge about medication after infection in pregnant women and newborns. One participant said:

*“I browsed my phone again and again. What is the effect of infection on newborns? What should pregnant women do? Which medicine can the baby or pregnant women take? Take a look.” (P3)*.

### Growth and gains as pregnant mother

Pregnant nurses experienced several challenges while working during the pandemic. Nevertheless, they also experienced growth and gains through this process.

#### Being a mature person

Being a mother is a new identity for the pregnant nurse, this role is associated with more responsibilities. Thus, some participants realized that they were more mature than before.

*“Although I was young*,* I may have become a bit calmer as a mother.” (P5)*.

*“I didn’t think about things in the long run before. But now*,* I had to say*,* I started to make my career plan*,* aiming to take the postgraduate entrance exam.” (P11)*.

Furthermore, pregnant nurses needed to work and undergo prenatal examinations, while some even had to take care of their other children. Coordinating their own time is also a technical task, and their time-management skills have improved.

*“To take care of my children*,* I am now a master of time management*,* I feel.” (P7)*.

#### Obtaining support from others

Numerous participants mentioned that they received some support from others. Firstly, support from the head nurse was very important, some participants stated that their leaders arranged easy work for them during pregnancy.

*“The head nurse asked me about the scheduling. And the leader took care of me*,* my work was relatively a bit easier.” (P4)*.

Secondly, receiving help from colleagues during pregnancy was relatively common, especially in hospitals with a supportive working environment.

*“Everyone was very willing to help you. There is no such thing as being jealous of you or giving you cold words based on your easy work.” (P7)*.

From another aspect, communicating with colleagues can help the pregnant nurse to feel less lonely and gain some knowledge on pregnancy.

*“We would sit together and exchange some ideas*,* such as what pregnancy-related things to buy*,* and what we need to pay attention to during pregnancy. Sometimes*,* some senior female colleagues would give us some guidance based on their personal experience.” (P3)*.

Furthermore, family members of pregnant nurses provided more support, enabling the participants to live and work better.

*“I think my good mentality was mainly because of family support.” (P5)*.

## Discussion

To the best of our knowledge, this is the first qualitative study focusing on exploring the experiences of working as a pregnant clinical nurse during the COVID-19 pandemic. The findings of this study may help the nursing managers and researchers to understand the working experience of pregnant nurses during the pandemic, provide guidance for cultivating a healthy workplace environment for pregnant nurses, and provide direction for the development of needs-based intervention in the future.

“Being particularly difficult to ask for leave during special period,” one subtheme of theme 1 (“Still adhering to work as a clinical nurse despite being pregnant during the pandemic”) reflects how the nursing shortage in clinical practice and lack of a comprehensive personnel reserve utilization mechanism in major infectious diseases hindered pregnant nurses ask for leave smoothly. To increase the coping ability to the similar situation, mobilize human resources quickly and respond successfully, a hospital-based emergency response mechanism for emerging major infectious diseases should be established. Hospital readiness checklist for COVID-19 recommend by the World Health Organization was shown as an example [41]. Meanwhile, specific guidelines regarding how pregnant health care professionals, including nurses, face contagious diseases like COVID-19, should be developed. An instance from American College of Obstetricians and Gynecologists presents as “health care facilities may consider limiting exposure of the pregnant health care professionals to patients with confirmed/suspected COVID-19 infection, especially during high-risk procedures.” [42] To address the complicated, inflexible, and cumbersome application procedure for leave, an effective online leave management system is suggested.

“Afraid of being labelled as someone not dedicated to nursing profession” pushed our participants to work at frontline. Those participants mentioned further that they may face self-moral condemnation to ask for leave when the others are busy or other pregnant colleagues stick to work. This phenomenon manifests the moral stigma, one aspect of maternity-leave stigma [43], and can be partly attributed to the Confucian cultural value [44, 45]. As a multi-dimensional concept, the maternity-leave stigma among pregnant nurses during COVID-19 should be explored further [43]. Thus, to build a fertility-friendly environment.

Theme 2 “Working during pregnancy under pandemic is still an ordinary nurse” indicates the same work content, same job requirements, and same COVID-19 related care as other nonpregnant nurses. A previous study from Korea which involved 12 shift work nurses who had experienced pregnancy within three years also found similar phenomenon [25]. The perception that “pregnancy is not an illness,” [46] combined with the challenges of the COVID-19 pandemic, further exacerbated the negative phenomenon of assigning the same workload to pregnant nurses.

It should be noted that some nursing tasks might pose a threat to pregnant nurses, especially during the COVID-19 pandemic. For example, caring for those with infectious disease like COVID-19 which can be transmitted from the mother to the fetus and the neonate [47], and having potential risk to pregnant women/fetus from prolonged N95 use (e.g., hindering gas exchange and placing extra strain on the metabolic system of pregnant women) [48, 49], working overtime, and practicing unsuitable nursing tasks during pregnancy (e.g., performing CPR, giving medications which could cross the placental barrier) [2], and assisting with patient movement [2]. It is an urgent necessity to create a work environment that guarantees safe continued pregnancy, and the important step is to seek to create and enforce policies that require healthcare work environments to study and address occupational safety health of nurses during pregnancy. Developing corresponding manuals targeting pregnant workers and establishing training programs for those nurses planning to become pregnant are two ways to achieve this goal. In the manual, it should recommend clearly which tasks pregnant nurses are safe to practice, and which ones that could affect and should be avoided [26]. If eliminating work environment hazards by reassigning to another position or other duties is impossible, laws similar to those in Quebec, which allow for preventative withdrawal, can serve as an example to enhance the occupational health and safety of pregnant nurses while encouraging workplaces to implement necessary changes to safeguard these employees [2].

Working as a pregnant nurse means experiencing “duelling roles” [50]. With experience of being disturbed by symptoms induced by pregnancy and experience of fulfilling nursing duties that put themselves and their baby at risk made participants more aware of their future role as mother (theme 3.3 still staying in the special life phase as a pregnant mother), instead of nurses. Those uncomfortable symptoms and occupational hazards might cause challenges in practicing nursing care. Future explorations on how to ensure the safety and quality of nursing care provided by pregnant nurses, especially during the emerging infectious diseases period, are warranted.

Although our study demonstrated that pregnant nurses adopted approaches to protect themselves and their upcoming babies very cautiously, most of them remained confused about the suitableness and usefulness of their chosen methods. For instance, washing many times until skin peeled after contacting those with scabies, and wearing radiation resistant clothing. Meanwhile, same as the finding showing the dilemma about taking the COVID-19 vaccine [50], our participants were confused about the potential impacts of the COVID-19 and related vaccinations on the fetuses. This uncertainty may be induced by lacking information and training on standard protection during pregnancy as well as lacking knowledge regrading COVID-19 and the vaccine on pregnant women and fetuses. Thereby, it is a necessity to develop self-protection interventions during pregnancy, especially those targeting the newly emerging infectious disease. The educational intervention on the basis of protection motivation theory, focusing on pregnant women’s self-protection and knowledge about COVID-19, could serve as a reference [51]. Furthermore, the scientific information regarding COVID-19, its vaccination and booster on maternal-fetal outcomes should also be disseminated, thus, to reduce related anxiety [52].

Subtheme 3.3.3 “becoming sensitive, vulnerable and nervous” reflects the negative mental health status among pregnant nurses during the COVID-19 pandemic. This phenomenon might be related to workplace bullying, a common feature of the nursing culture, directed toward junior nurses by head nurses or senior nurses [53]. In a society with relatively strong hierarchy, even those disadvantaged like pregnant nurses, they should still follow the potential rules of “nurses with more years of experiences pregnant first”. If a novice nurse was pregnant first, she might experience blame or discrimination from other colleagues. As a fresh finding, more studies are needed to explore in-depth regarding bullying in nursing during pregnancy. Another cause to this negative mental health status was “feeling guilt due to bring trouble to other nurse coworkers.” Hino et al. confirmed similia findings, stating as “feeling bad about the burden that their pregnancies plane on other colleagues” [50]. Corresponding interventions aimed at reducing negative emotions, such as shame and guilt, should be developed. Acceptance and commitment therapy might be a potential potion [54].

The fourth theme refers to receiving gains as pregnant mother. The nurses in this study stated that experiencing pregnancy help them to be more mature. This aligns with the results of two previous studies [2, 25]. The first study demonstrated that experiencing pregnancy increased the sense of responsibility in mother nurses [25]. The second study described the mother nurses as “super nurses” [2]. Differently, our study did not show the positive impact as pregnant nurses in terms of professional growth [25]. In Lee et al.’ study, a participant expressed as “the families of patients gave me more trust, thinking that since I was a pregnant nurse in a pediatric unit, so I must take care of my patients very well.” [25]. None of the nurses in our study worked in pediatrics, gynecology or obstetrics might explain the differences.

Gaining support from managers and colleagues, the main facilitator to avoid occupational hazards during pregnancy [2], was also mentioned by some participants. This may be related to a healthy workplace environment. As shown, active management style, as well as constructive relationships with supportive supervisors and co-workers, may increase the resilience of nurses and their ability to work during pregnancy, enhance their job satisfaction and, thus, reduce the turnover rate [55]. More studies focusing on interventions to improve pregnant nurses’ work environments, such as using educational strategies, participatory approach, or accreditation process [56], are required.

### Limitation

Some limitations of this study should be noted. Firstly, all the participants were recruited from ten departments in seven hospitals in the same city in China, and all had at least bachelor’s degrees. The present findings could be further enriched by recruiting participants with varying educational backgrounds or those working in other departments (e.g., pediatrics, gynecology, obstetrics), or in hospitals from different regions of China. Secondly, semi-structed interviews were conducted online; this approach is potentially limited by the inability to observe the whole behavioral language [57].

## Conclusion

This study explored the experience of working as a pregnant clinical nurse during the COVID-19 pandemic. Pregnant clinical nurses experienced various changes and difficulties during the pandemic. Meanwhile, they also experienced growth and gains after this experience. Future studies on creating a work environment that guarantees safe continued pregnancy, especially during the emerging infectious diseases, are required. Such actions should focus on the development of specific guidelines and manuals regarding how pregnant nurses work, as well as the establishment of self-protection interventions during pregnancy. Furthermore, studies should explore the phenomenon of moral stigma and bullying in nursing during pregnancy.

## Data Availability

Data are available on request from the authors. The data that support the findings of this study are available from the corresponding author upon reasonable request.
